# Defining the consequences of endogenous genetic variation within a novel family of *Schizosaccharomyces pombe* heterochromatin nucleating sequences

**DOI:** 10.1093/g3journal/jkab185

**Published:** 2021-06-02

**Authors:** Arati Joshi, Meryl J Musicante, Bayly S Wheeler

**Affiliations:** Department of Biology, Rhodes College, Memphis, TN 38112, USA

**Keywords:** centromere, heterochromatin, repetitive DNA, fission yeast, chromatin

## Abstract

Centromeres are essential for genetic inheritance—they prevent aneuploidy by providing a physical link between DNA and chromosome segregation machinery. In many organisms, centromeres form at sites of repetitive DNAs that help establish the chromatin architecture required for centromere function. These repeats are often rapidly evolving and subject to homogenization, which causes the expansion of novel repeats and sequence turnover. Thus, centromere sequence varies between individuals and across species. This variation can affect centromere function. We utilized *Schizosaccharomyces pombe* to assess the relationship between centromere sequence and chromatin structure and determine how sensitive this relationship is to genetic variation. In *S. pombe*, nucleating sequences within centromere repeats recruit heterochromatin via multiple mechanisms, which include RNA-interference (RNAi) . Heterochromatin, in turn, contributes to centromere function through its participation in three essential processes; establishment of a kinetochore, cohesion of sister chromatids, and suppression of recombination. Here, we show that a centromere element containing *RevCen*, a target of the RNAi pathway, establishes heterochromatin and gene silencing when relocated to a chromosome arm. Within this *RevCen*-containing element (RCE), a highly conserved domain is necessary for full heterochromatin nucleation but cannot establish heterochromatin independently. We characterize the 10 unique RCEs in the *S. pombe* centromere assembly, which range from 60% to 99.6% identical, and show that all are sufficient to establish heterochromatin. These data affirm the importance of centromere repeats in establishing heterochromatin and suggest there is flexibility within the sequences that mediate this process. Such flexibility may preserve centromere function despite the rapid evolution of centromere repeats.

## Introduction

Centromeres are essential for the transmission of eukaryotic genomes through cell division. The centromere directs kinetochore assembly and works with it to connect chromosomes to mitotic and meiotic spindles. Defects in centromere structure can cause chromosome missegregation, aneuploidy, and genome instability (Thompson *et al.* 2010). Across diverse organisms, centromeres share a common epigenetic architecture that is the basis of centromere function: chromatin containing the centromere-specific histone variant CENP-A is flanked by heterochromatin, marked by methylated histone H3 lysine 9 (H3K9me) (McKinley and Cheeseman 2016). CENP-A recruits additional centromere and kinetochore proteins (Blower and Karpen 2001; Black *et al.* 2007; Mendiburo *et al.* 2011), and its loss causes chromosome missegregation, cell cycle arrest, and cell death (Stoler *et al.* 1995; Buchwitz *et al.* 1999; Howman *et al.* 2000; Blower and Karpen 2001). H3K9me-enriched pericentromeric heterochromatin recruits cohesins and thus supports chromosome segregation by promoting the attachment of sister chromatids and preventing their premature segregation (Kellum and Alberts 1995; Bernard *et al.* 2001; Thompson *et al.* 2010; Hahn *et al.* 2013; Yi *et al.* 2018). Pericentromeric heterochromatin also facilitates appropriate chromosome segregation by blocking centromere-proximal meiotic crossovers, which are associated with chromosome nondisjunction during gamete development (Slatis 1955; Koehler *et al.* 1996; Ellermeier *et al.* 2010).

Centromeres often form over repetitive DNA sequences. These repeats play an important role in centromere function by establishing the requisite chromatin architecture (Ohzeki *et al.* 2002; Partridge *et al.* 2002; Catania *et al.* 2015; Fachinetti *et al.* 2015). Variation among centromere repeats is consequential, and some naturally occurring variants are associated with chromosome missegregation in humans (Fachinetti *et al.* 2015; Dumont *et al.* 2020). Although centromere repeats contribute to centromere function, they are also rapidly evolving and diverge between species (Hartley and O’Neill 2019). Defining how centromere repeats contribute to chromatin formation and the extent to which variation among these sequences influences centromere structure is critical to understanding how organisms withstand the rapid evolution of DNA sequences at the centromere.

The fission yeast *Schizosaccharomyces pombe* is an ideal system in which to define the relationship between centromeric chromatin and its underlying repetitive DNA sequences. Their compact centromeres and well-elucidated chromatin assembly pathways enable the association of individual sequences with their function (Allshire and Ekwall 2015). Each of the three *S. pombe* centromeres are organized around a central core and a pair of innermost repeats that are enriched in the *S. pombe* CENP-A homolog, Cnp1 (Clarke and Baum 1990; Takahashi *et al.* 2000). Cnp1-enriched sequences are surrounded by pairs of *dg* and *dh* repeats that make up the outer repeats (*otr*). *dg* and *dh* are enriched in heterochromatin, as indicated by H3K9me2, H3K9me3, and the HP1 homolog Swi6 (Nakaseko *et al.* 1986; Chikashige *et al.* 1989; Nakayama *et al.* 2001). In fission yeast, heterochromatin plays an important role in all aspects of centromere function; heterochromatin loads Cnp1, recruits cohesins, and suppresses recombination (Bernard *et al.* 2001; Folco *et al.* 2008; Ellermeier *et al.* 2010). Although heterochromatin is enriched across *dg* and *dh* (Cam *et al.* 2005), only a subset of sequences within *dg* and *dh* are capable of establishing heterochromatin *de novo* (Partridge *et al.* 2002; Buscaino *et al.* 2013; Marina *et al.* 2013; Parsa *et al.* 2018). Once heterochromatin is assembled at these nucleation sequences, it spreads into adjacent nonnucleating sequences (Buscaino *et al.* 2013). Because both nucleating and nonnucleating sequences are enriched in H3K9me2 and Swi6, the presence of heterochromatin is insufficient to distinguish between them. Canonically, nucleating sequences have been identified based on their ability to establish heterochromatin *de novo* on artificial chromosomes or at ectopic chromosomal sites (Hall *et al.* 2002; Partridge *et al.* 2002; Buscaino *et al.* 2013; Marina *et al.* 2013; Parsa *et al.* 2018). The ability of sequences to nucleate heterochromatin at an ectopic site reveals a sequence-dependent mechanism of heterochromatin establishment that can be distinguished from the sequence-independent mechanisms (spreading and the epigenetic inheritance of existing heterochromatin) that shape heterochromatin in the context of the centromere (Allshire and Ekwall 2015).

The recognition of nucleating sequences by the RNA-interference (RNAi) pathway is both necessary and sufficient for sequence-dependent nucleation (Volpe *et al.* 2002; Bühler *et al.* 2006). In the RNAi pathway, centromere transcripts are cleaved into siRNAs by the ribonuclease Dcr1 (Reinhart and Bartel 2002; Volpe *et al.* 2002). siRNAs are loaded onto effector complexes that are recruited to centromere repeats by base-pairing between the siRNA and homologous transcripts and by the recognition of existing H3K9me2 (Noma *et al.* 2004; Verdel *et al.* 2004; Irvine *et al.* 2006; Schalch *et al.* 2009). Once at the centromere, effector complexes recruit additional RNAi and heterochromatin proteins (Motamedi *et al.* 2004; Sugiyama *et al.* 2005). The number of siRNAs produced from a sequence at its centromeric location is correlated with its ability to nucleate heterochromatin (Buscaino *et al.* 2013). Two fragments of a *dg* repeat that are associated with high levels of siRNAs are capable of nucleating heterochromatin at an ectopic site, while a separate *dg* fragment that is associated with lower levels of siRNAs is unable to (Buscaino *et al.* 2013). Whether the relationship between siRNA production and nucleation applies more broadly to other nucleating sequences remains unknown.

Without a clear understanding of the features that define nucleating sequences, empirical approaches are required to identify the sequences that establish the epigenetic landscape required for a functional centromere. We focused our experiments on a 788-bp sequence element in the *dg* repeat of centromere one. Four lines of evidence suggested that this element may function as a heterochromatin nucleating sequence. First, a related sequence is contained within a 1.6 kb *dg* fragment that nucleates heterochromatin (Buscaino *et al.* 2013). Second, siRNAs homologous to this element are abundant in wild-type yeast (Djupedal *et al.* 2009). Third, this element contains a fragment called *RevCen* that is cleaved into siRNAs by Dcr1 *in vitro* (Djupedal *et al.* 2009). Finally, in addition to *RevCen*, this fragment contains a centromere promoter and intron, sequence features that have been associated with heterochromatin nucleation (Buscaino *et al.* 2013; Marina *et al.* 2013; Mutazono *et al.* 2017). Using an ectopic heterochromatin assay, we demonstrate that this *RevCen*-containing element (RCE) is sufficient to establish heterochromatin and gene silencing*.* This ability is dependent, in part, on a 329-bp sequence within the *RevCen* fragment. This sequence is shared between *dh* and *dg* repeats as a consequence of a translocation event (Chikashige *et al.* 1989), and, despite its distribution across repeats and chromosomes, remains well conserved. We identified additional RCEs containing this conserved sequence and show that, despite sequence variation up to 40%, all 10 unique RCEs are capable of establishing heterochromatin. These results define RCEs as a group of heterochromatin nucleating sequences, expanding the number of known nucleation-capable sequences, and demonstrate that the sequence-dependent pathways that target RCEs and establish heterochromatin domains are capable of recognizing a range of DNA sequences. Such flexibility may allow fission yeast to maintain centromere function despite the accumulation of new mutations.

## Materials and methods

### Plasmid construction

Plasmids used in this study were constructed via Gibson Assembly with PCR primers listed in Supplementary Table S1 (Gibson *et al.* 2009). Full-length RCEs were amplified from the genome of the fission yeast strain Kfy501. RCE(dg) elements were amplified via PCR with the primer pair WLP19F/R, which anneals to all known RCE(dg) elements except for RCE(2Ldg). RCE(2Ldg) was amplified with WLP19F/WLP19bR. RCE(dh) elements were amplified by PCR using WLP32F/R or WLP32F/WLP19R, depending on their downstream flanking sequences. Truncated versions of RCE(1Rdg) were amplified from a plasmid containing the full-length RCE(1Rdg) element using primers listed in Supplementary Table S1. PCR amplicons were inserted into the *Spe*I site of the plasmid BW5 using the HiFi assembly master mix (NEB) (Wheeler *et al.* 2009). Sanger sequencing determined the identity of the element within each plasmid.

### Fission yeast strains

The fission yeast strains used in this study are as listed (Supplementary Table S2). Fission yeast were grown using standard procedures (Moreno *et al.* 1991). To insert constructs at the *ura4* locus, plasmids were linearized by restriction digest and transformed into the *clr4Δ* strain Kfy976 or the *clr4^+^* strain Wfy18 via electroporation using a BioRad Micropulser set to the *Schizosaccharomyces* protocol (ShS). Yeast were plated on pombe minimal glutamate (PMG) media without adenine and incubated at 32°C for 5–7 days (Sunrise scientific). To select for yeast that integrated the construct within *ura4*, transformants were patched onto PMG media containing 1 g/L of 5-fluoroorotic acid (FOA) (Zymo research). FOA-resistant colonies were further screened for appropriate construct integration by PCR. Strains resulting from transformation into the *clr4Δ* host strain Kfy976 were crossed to the *clr4^+^* strain Wfy18 before analysis. Three independent transformants of each genotype were used as biological replicates for all experiments.

### *ade6* ^+^ expression assays

For serial dilution assays, strains were grown in YES media (yeast extract with supplements) overnight at 32°C with shaking. Cultures were diluted to a concentration of 1 × 10^6^ cells/mL, and a 10-fold dilution series was created for each strain. For each dilution, 5 µL were plated on PMG media with limiting (1/10th) adenine and incubated for four nights at 32°C and one night at 4°C before photographing. To measure the percentage of *RCcons-ade6^+^* and *RCE(ΔRCcons)-ade6^+^* yeast that exhibited silencing, three biological replicates of each genotype were grown overnight in YES. Cultures were plated on PMG with 1/10th adenine and grown for four nights at 32°C and one night at 4°C before photographing. Colony color was interrogated by eye, and white colonies were distinguished from those with evidence of silencing, a group that includes red, pink, and sectored colonies. This process was then repeated for cultures derived from red *RCE(ΔRCcons)-ade6^+^* colonies.

### Chromatin immunoprecipitation

Chromatin immunoprecipitation (ChIP) was performed as described with the following modifications (Cam and Whitehall 2016). For each construct, three independent transformants were grown in 100 mL of YES to a density between 8 × 10^6^ and 2 × 10^7^ cells/mL. Paraformaldehyde was added to each culture to achieve a final concentration of 2.7%. Cultures were fixed for 15 minutes at room temperature with intermittent shaking, and 5 mL of 2.5 M glycine was added to stop fixation. Fixed cells were washed three times withtris-buffered saline before freezing on dry ice. After beadbeating, chromatin was sonicated using a Qsonica Q125 probe sonicator set to an amplitude of 30%. Samples were sonicated for four rounds of six 10-second pulses. This shearing protocol resulted in an average fragment size of ∼400 bp. Sheared chromatin was precleared via a 1- to 2-hour incubation with protein A/G magnetic beads (Pierce) before dividing preps into input and IP fractions. IPs were incubated overnight with 1 µL of anti-H3K9me2 serum (Active Motif 39376) and incubated for 1–2 hours in the presence of protein A/G magnetic beads. After washing the beads, IP and input crosslinks were reversed overnight, and DNA from input and IP samples was isolated using magnetic beads with a ratio of 1.8:1 beads: sample (MAGBIO).

### Quantitative PCR

ChIP enrichment was determined using a BioRad CFX96 qPCR machine and Luna SYBR green mastermix (NEB). Input ChIP samples were diluted 1:100 and 1 µL of diluted input or undiluted IP were added to each 10 µL PCR reaction. Each PCR reaction was performed in triplicate. For every primer pair in every PCR, a fivefold serial dilution of genomic DNA was included to calculate the PCR efficiency. PCRs included in this study had efficiencies between 90% and 110% with a correlation coefficient >0.9. Primer pairs were validated to produce a single melt-curve peak indicative of a single PCR product. Enrichment was calculated by dividing the mean starting quantity of query DNA by the mean starting quantity of *act1* DNA for both input and IP samples. The normalized enrichment reported here was calculated as the IP enrichment divided by the input enrichment. Means of at least three biological replicates were plotted along with the SEM. Tests of statistical significance were conducted using an unpaired *t*-test assuming a Gaussian distribution (Prism 8). Uncorrected *P*-values are reported.

### Data availability

Strains, plasmids, and oligos are available upon request. Supplementary Table S1 contains primers used to construct each plasmid, and Supplementary Table S2 contains the genotypes of all strains used in this study. Supplementary material is available at figshare: https://doi.org/10.25387/g3.14444531.

## Results

### A RCE is capable of establishing heterochromatin and gene silencing

To identify nucleating sequences in *S. pombe*, we used an established ectopic heterochromatin assay (Wheeler *et al.* 2009). In this assay, potential nucleating sequences are integrated at the *ura4* locus, where their ability to establish gene silencing and heterochromatin can be measured. Potential nucleating sequences are inserted adjacent to an *ade6^+^* reporter gene, which allows gene silencing to be observed visually. When *ade6^+^* is expressed, colonies are white, and when *ade6^+^* is silenced, colonies are red. The formation of heterochromatin at *ura4*, a normally euchromatic site that supports ectopic heterochromatin (Wheeler *et al.* 2009), is measured via chromatin immunoprecipitation with antibodies against H3K9me2. H3K9me2 is a hallmark of heterochromatin in *S. pombe* (Nakayama *et al.* 2001), and H3K9me2 levels at the ectopic site are correlated with the levels of the heterochromatin protein and HP1 homolog Swi6 (Wheeler *et al.* 2009).

**Figure 1:**
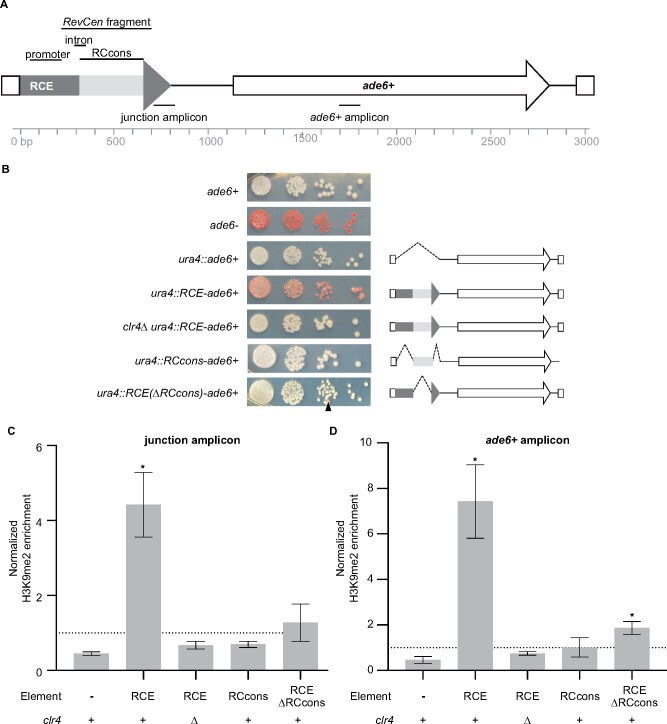
A *RevCen*-containing element silences gene expression and establishes heterochromatin through a mechanism that depends on Clr4 and the *RevCen* conserved domain. (A) The construct integrated at *ura4* contained an RCE from the right side of centromere 1 and an *ade6^+^* reporter gene. Regulatory sequences within the RCE are indicated above the schematic (Chikashige *et al.* 1989; Djupedal *et al.* 2005, 2009; Mutazono *et al.* 2017). H3K9me2 enrichment was interrogated at two loci, indicated below the schematics, by ChIP-qPCR. (B) The RCE silences *ade6^+^ ade6^+^* expression was assessed using a serial dilution assay. Each row contains a single strain plated on adenine-limiting media. The genotype of each strain is listed to the left of the assay, and a schematic of each construct is indicated to the right. *ade6^+^* (white) and *ade6^−^* (red) colonies demonstrate the phenotypic effects of *ade6^+^* expression and are compared to a representative *ura4::ade6^+^* control strain. The arrowhead indicates the presence of silenced colonies in *ura4::RCE(ΔRCcons)-ade6^+^* strains. The RCE establishes heterochromatin locally (C) and at the *ade6^+^* reporter gene (D). *X* axis labels indicate the element included within the construct and whether Clr4 was present in each strain. Normalized enrichment was calculated as qPCR signal for immunoprecipitated relative to input samples at the query loci *vs* the control *act1^+^* locus. Average normalized enrichment, as calculated from three or more replicates, is indicated as gray bars. Error bars depict the SEM. The dotted line at *y* = 1 represents the expected enrichment in the absence of H3K9me2. Asterisks indicate a significant difference between the relevant strains and a *clr4Δ ura4::RCE-ade6^+^* control (*P* ≤ 0.05, one asterisk). *P*-values were calculated using an unpaired Student’s *t*-test assuming a Gaussian distribution (Prism 8). *P*-values derived from additional comparisons are presented in the Supplementary Tables S3 and S4.

Within the *dg* repeat of centromere I, the 788-bp RCE contains a promoter, an intron, and the *RevCen* fragment that is targeted by Dcr1 *in vitro* (Figure 1A) (Djupedal *et al.* 2005, 2009; Mutazono *et al.* 2017). As transcription, splicing, and Dcr1-mediated cleavage are important for heterochromatin establishment by other sequences (Volpe *et al.* 2003; Buscaino *et al.* 2013; Marina *et al.* 2013; Mutazono *et al.* 2017), we hypothesized that the RCE may function as a heterochromatin nucleating sequence. Our data support that hypothesis. When the RCE is at the ectopic site, colonies exhibit a variegated morphology (Figure 1B), and H3K9me2 is significantly enriched at the RCE and at *ade6^+^* (junction H3K9me2 fold enrichment = 4.4, *P* = 0.01; *ade6^+^* fold enrichment = 7.4, *P* = 0.01) (Figure 1, C and D; Supplementary Tables S3 and S4). H3K9me2 enrichment is lower at the ectopic site than at an endogenous *dg* repeat, where we observe an average 43-fold enrichment (FE), and slightly lower than the levels observed when the nucleating sequence L5 is at the ectopic site (junction H3K9me2 FE = 7.8; *ade6^+^* FE = 9.1) (Wheeler *et al.* 2009). Silencing and H3K9me2 enrichment are dependent on the RCE and on an intact heterochromatin assembly pathway; in the absence of the RCE or Clr4 (the sole H3K9me methyltransferase in *S. pombe*) *ade6^+^* does not become silenced and H3K9me2 is undetectable (Figure 1, B–D).

### The *RevCen* conserved domain contributes to but is not sufficient for the nucleation of heterochromatin

Given the abundance and conservation of the *RevCen* conserved domain, we hypothesized that it may play a role in heterochromatin formation. We show that the *RevCen* conserved domain is not sufficient for heterochromatin establishment or gene silencing. When this sequence is integrated upstream of *ade6^+^*, colonies retain the *ade6^+^* expressed phenotype and H3K9me2 is not enriched at the ectopic site (Figure 1, B–D; Supplementary Figure S1). However, the *RevCen* conserved domain does contribute to the nucleation of heterochromatin by the RCE. Most *RCE(ΔRCcons)-ade6^+^* colonies exhibit the *ade6^+^* expressed phenotype, and *RCE(ΔRCcons)-ade6^+^* yeast exhibit a significant reduction in H3K9me2 at the ectopic site as compared to *RCE-ade6^+^* yeast (junction fold change  = 0.16, *P* = 0.01; *ade6^+^* fold change = 0.25, *P* = 0.03) (Figure 1, B–D; Supplementary Figure S1). Surprisingly, the sequences surrounding the *RevCen* conserved domain have the capacity to nucleate heterochromatin independently, as evidenced by the observations that *ade6*-silenced colonies can be detected in *RCE(ΔRCcons)-ade6^+^* strains, (Figure 1B, arrowhead; Supplementary Figure S1) and that a low but significant level of H3K9me2 enrichment can be detected at *ade6^+^* in the presence of RCE(ΔRCcons) (FE = 1.88, *P* = 0.02) (Figure 1D). To quantify the extent of silencing in *RCE(ΔRCcons)-ade6^+^* strains, we plated yeast on adenine-limiting media and counted the number of colonies with visible evidence of *ade6^+^* silencing. We found that 4.9% of colonies derived from randomly selected *RCE(ΔRCcons)-ade6^+^* yeast exhibit silencing in comparison to 0% of colonies containing only the *RevCen* conserved domain at the ectopic site (Supplementary Figure S1). Silencing is partially maintained in *RCE(ΔRCcons)-ade6^+^* progeny; 53% of silenced-derived colonies exhibit silencing upon replating (Supplementary Figure S1). Together, these results highlight the interdependent relationship between the *RevCen* conserved domain and the rest of the RCE: the flanking sequences are sufficient for nucleating a low level of heterochromatin, but the conserved domain is necessary for robust silencing and heterochromatin nucleation.

### All centromeric RCEs act as heterochromatin nucleating sequences

**Figure 2:**
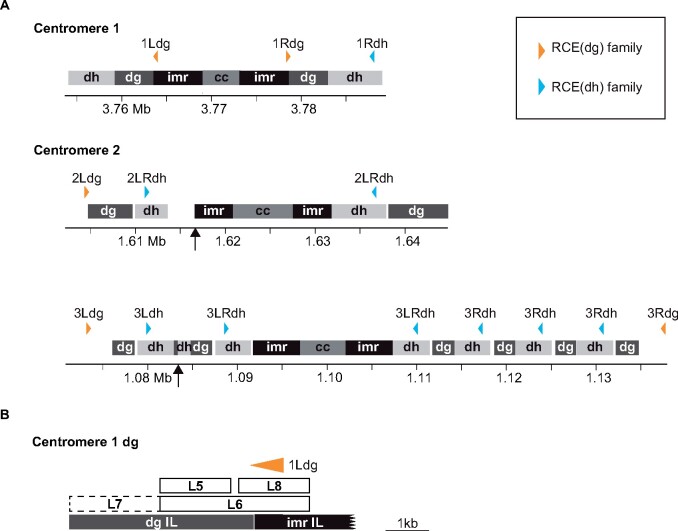
*RevCen*-containing elements are distributed among all three *S. pombe* centromeres. (A) Schematic representations of the three centromeres are shown to scale. The two major heterochromatin repeats, *dg* and *dh*, shown in dark and light gray, respectively, flank the innermost repeats (imr) and central core (cc). Above these sequences, the location of individual RCEs is indicated by a colored triangle and a label. The color indicates the subfamily to which the element belongs; RCE(dg) members are shown in orange and RCE(dh) members are shown in light blue. The label indicates the heterochromatin domain (right or left side of a specific centromere), and RCEs that have identical sequences are given the same name. Arrows below the centromeres indicate gaps in the centromere assembly. (B) The *dg* repeat from centromere 1L is shown here. Fragments with nucleating capacity, including L5, are indicated by a solid outline (Partridge *et al.* 2002; Buscaino *et al.* 2013). L7, a fragment without detectable nucleating capacity, is indicated by a dashed outline. RCE(1Ldg) is shown as a triangle.

The *RevCen* conserved domain is found in 14 copies in the *S. pombe* centromere assembly. These copies range from 93% to 100% identical (EMBOSS Needle; Madeira *et al.* 2019). The ubiquity of this sequence is the result of its translocation between *dh* and *dg* repeats (Chikashige *et al.* 1989). As such, the *RevCen* conserved domain is found in different contexts, and the flanking sequences can be used to divide RCEs into two subfamilies. To refer to individual RCEs, we use the format RCE(1Ldg) where the information in parentheses indicates the subfamily (dg) and the location (the left side of chromosome 1). Members with the same sequence share a name: for example, RCE(2LRdh) is present in identical copies on the left and right sides of chromosome 2. We identified five unique members of the first subfamily, which we named RCE(dg) for their shared proximity to *dg* repeats (Figure 2A; Supplementary Table S5). RCE(dg) contains the founding RCE characterized in Figure 1. Hereafter, we refer to this RCE as RCE(1Rdg) to distinguish it from related elements. RCE(1Ldg) is found within a larger fragment, L8, that nucleates heterochromatin (Figure 2B) (Buscaino *et al.* 2013). We named the second subfamily RCE(dh) as all members are found within *dh* repeats. There are five unique members of RCE(dh) found in nine copies in the current centromere assembly (Figure 2A; Supplementary Table S5). When comparing across the entire element, including conserved and flanking sequences, centromeric RCEs range from 60% to 100% identical (EMBOSS Needle; Madeira *et al.* 2019). One additional member of RCE(dh) resides outside of the centromere within the heterochromatic mating-type locus. In addition to the 14 RCEs in the centromere assembly, there are likely additional RCEs present in the *S. pombe* genome that are absent from the assembly. Sequence gaps on chromosome 2 and chromosome 3 contain missing *dg*/*dh* repeats and, likely, additional RCEs (Figure 2A, arrows).

**Figure 3:**
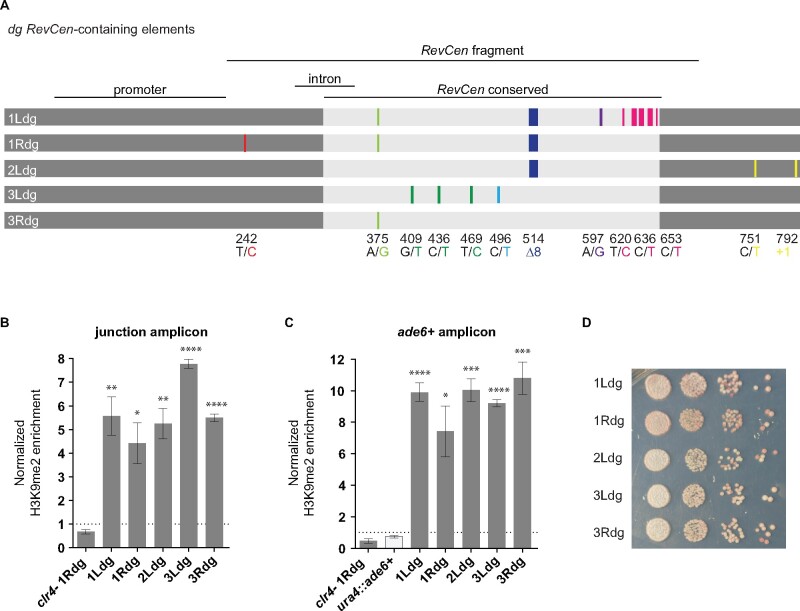
All RCE(dg) members function as heterochromatin nucleating sequences. (A) The sequences of the five unique RCE(dg) members are shown as individual bars. The positions of known sequence elements are indicated and named above the bars. The color at each position within the bars indicates whether the corresponding base matches (gray) or differs from (color) a consensus sequence. Bases in light gray match the *RevCen* conserved domain consensus sequence, which was built by comparing all copies present in the centromere assembly. Outside of the *RevCen* conserved domain, bases in dark gray match the RCE(dg) consensus sequence. Variants that co-occur in the assembly are shown in the same color. Consensus (black) and alternate (color) bases are listed along with their position below the bars in line with the variant. RCE(1Ldg) contains a cluster of eight variants; only three are named here for simplicity. H3K9me2 levels were interrogated at the relevant RCE(dg) member (B) and at *ade6^+^* (C). Average enrichment for at least three biological replicates is shown along with error bars that represent the SEM. An unpaired *t*-test was used to compare ectopic H3K9me2 in the indicated strain to ectopic H3K9me2 in a *clr4Δ ura4::RCE(1Rdg)-ade6^+^* control. *P*-values are represented by asterisks: *P* ≤ 0.05, one asterisk; *P* ≤ 0.01, two asterisks; *P* ≤ 0.001, three asterisks; *P* ≤ 0.0001, four asterisks. Comparisons among RCE(dg) members are included in Supplementary Tables S6 and S7. (D) Strains containing RCE(dg) members at the ectopic site were diluted and plated on adenine-limiting media. Red, pink, and sectored colonies are indicative of *ade6^+^* silencing.

To determine whether all members of RCE(dg) are capable of establishing heterochromatin, we integrated each member at the ectopic *ura4* site, along with the *ade6^+^* reporter gene. In each case, we included the promoter, intron, and *RevCen* fragment in the construct (Figure 3A). Both the promoter and the intron are perfectly conserved across all members of RCE(dg) (Figure 3A). As with previous experiments, we measured heterochromatin enrichment at the RCE and at *ade6^+^*. Our data show that all RCE(dg) members are capable of nucleating heterochromatin at the *ura4* locus; in the presence of individual RCEs, H3K9me2 is significantly enriched relative to a *clr4Δ ura4::RCE(1Rdg)-ade6^+^* control (*P* < 0.05) (Figure 3, B and C). RCE(dg) members recruit similar levels of H3K9me2 to the ectopic site (junction FE range = 4.4–7.8; *ade6^+^* FE range = 7.4–10.8), and we detect relatively few significant differences among them (Supplementary Tables S6 and S7). Locally, RCE(3Ldg) recruits more H3K9me2 than many RCE(dg) members (fold change comparing RCE(3Ldg) to all other RCE(dg) in aggregate = 1.5, *P* = 0.0033) (Supplementary Table S6). However, the elevated levels of H3K9me2 are restricted to the RCE(3Ldg) junction and are not observed at *ade6^+^* (Supplementary Table S7). In addition to recruiting H3K9me2, all RCE(dg) members are capable of silencing *ade6^+^*, as indicated by the presence of red, pink, and sectored colonies when RCE(dg) members are integrated at the ectopic site (Figure 3D).

**Figure 4:**
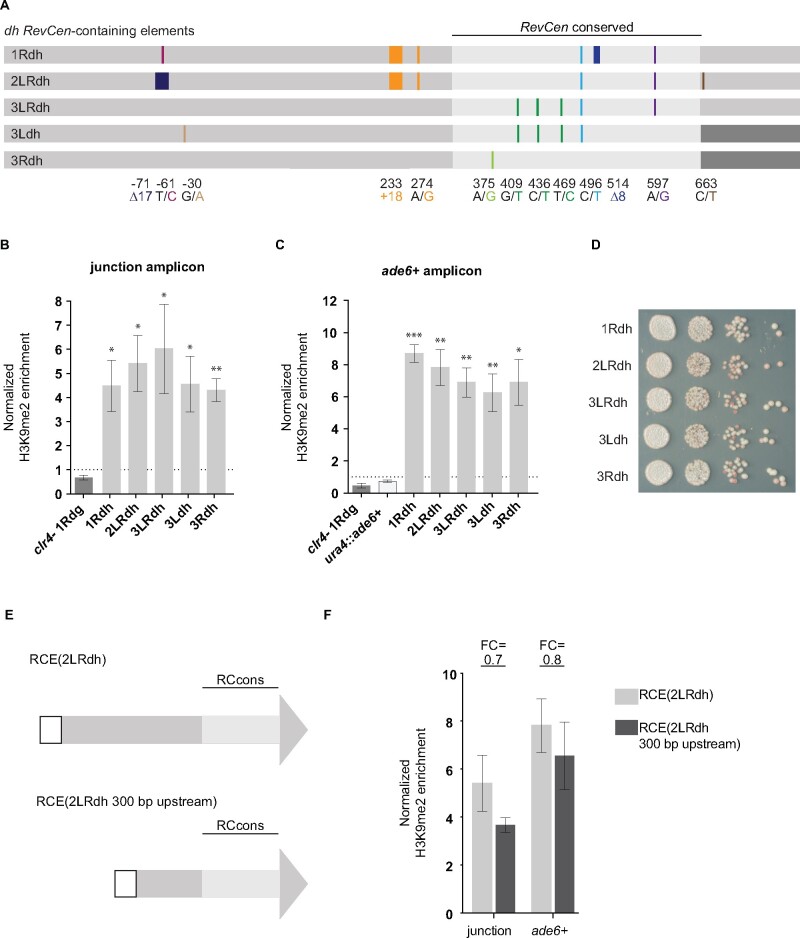
All RCE(dh) members function as heterochromatin nucleating sequences. (A) RCE(dh) family members are shown in colors consistent with those in Figure 3. The variant position is also consistent with Figure 3. Bases shaded in an intermediate gray match the RCE(dh) consensus sequence. The downstream flanking sequences of two RCE(dh) elements, RCE(3Ldh) and RCE(3Rdh), match the RCE(dg) consensus; these sequences are shaded in dark gray. As with Figure 3, H3K9me2 levels were interrogated at the relevant RCE(dh) element (B) and within the *ade6^+^* reporter gene (C). (D) Strains containing RCE(dh) members at the ectopic site were plated on adenine limiting media to resolve *ade6^+^* expression (E) Diagrams depicting RCE(2LRdh) and a truncated version of the element, including 300-bp upstream of the *RevCen* conserved domain, are shown here. (F) H3K9me2 levels were determined as with previous figures and are shown in light gray for strains containing the full-length RCE(2LRdh) and in dark gray for strains with300 bp of upstream DNA. The fold change in H3K9me2 levels is indicated above each pair of bars.

We next examined whether all members of RCE(dh) could similarly establish heterochromatin. Within RCE(dh), the sequences upstream of the *RevCen* conserved domain are well-conserved but distinct from those in RCE(dg), a consequence of their independent origins (Figure 4A). RCE(dg) and RCE(dh) upstream sequences are 39% percent identical (pairwise comparison using EMBOSS Needle; Madeira *et al.* 2019). It is unknown whether RCE(dh) upstream sequences contain a promoter and they appear to lack an intron (Lee *et al.* 2013). Because of the importance of the flanking sequences in RCE(1Rdg) and the dissimilarity between the RCE(dg) promoter and the corresponding sequences in RCE(dh), we included an additional 280 bp of upstream sequence in RCE(dh) constructs (Figure 4A). We find that all members of RCE(dh) are capable of establishing heterochromatin. In the presence of individual family members, H3K9me2 is significantly enriched relative to a *clr4Δ ura4::RCE(1Rdg)-ade6^+^* control (*P* < 0.05) (Figure 4, B and C). We fail to detect any significant differences in the levels of H3K9me2 recruited by different RCE(dh) members (junction FE range = 4.3–6.0; *ade6^+^* FE range = 6.3–8.7) (Supplementary Tables S6 and S7). We next compared the ability of RCE(dg) and RCE(dh) elements to nucleate heterochromatin. In aggregate, we find no significant difference in the ability of RCE(dg) and RCE(dh) members to recruit heterochromatin locally. However, at *ade6^+^*, H3K9me2 levels are slightly elevated in the presence of RCE(dg) members as compared to those in the presence of RCE(dh) members (fold change comparing all RCE(dg) to all RCE(dh) in aggregate = 1.3, *P* = 0.0057). As with RCE(dg), RCE(dh) family members silence *ade6^+^* expression when integrated at the ectopic site (Figure 4D).

To determine whether the additional upstream sequence included within RCE(dh) constructs was necessary for their ability to nucleate heterochromatin, we created strains that contain RCE(2LRdh) with only 300-bp upstream of the conserved sequences, which more resembles the 320 bp included in RCE(dg) (Figure 4E). We find that the truncated RCE(2LRdh 300 bp upstream) is able to nucleate heterochromatin to a similar extent as RCE(2LRdh) (junction fold change = 0.7, *P* = 0.27; *ade6^+^* fold change = 0.8, *P* = 0.51) (Figure 4F).

Together, these experiments demonstrate that members of both RCE(dg) and RCE(dh) are sufficient to nucleate heterochromatin and silence gene expression, despite varying in DNA sequence by as much as 40%.

## Discussion

### Centromere sequence, variation, and function

While centromere structure and function are widely conserved, the repeats over which centromeres form evolve at faster-than-neutral rates and diverge between closely related species (Hartley and O’Neill 2019). One resolution to this paradox suggests that it is the inheritance of chromatin, and not DNA sequence, that defines a centromere (Henikoff *et al.* 2001). However, studies of endogenous centromeres, in conjunction with findings from artificial chromosome research, have demonstrated that centromere function reflects the collective effort of sequence-dependent and sequence-independent pathways (Harrington *et al.* 1997; Grimes *et al.* 2002; Ohzeki *et al.* 2002; Barnhart *et al.* 2011; Hayden *et al.* 2013; Fachinetti *et al.* 2015; Logsdon *et al.* 2019). In fission yeast, centromere repeats are not required for centromere function (Ishii *et al.* 2008), but they do establish the requisite chromatin landscape at endogenous centromeres (Partridge *et al.* 2002; Catania *et al.* 2015). The work presented here contributes to our understanding of the relationship between centromere sequence and structure and invokes a model in which flexibility and redundancy allow centromere function to remain resilient as centromere repeats experience mutation and turnover.

### Flexible but sequence-dependent heterochromatin formation

Our data demonstrate the importance of DNA sequences within the *S. pombe* centromere*.* Short, discrete RCEs are sufficient for establishing heterochromatin and gene silencing when relocated a chromosome arm. Within RCE(dg), the sequences flanking the *RevCen*-conserved domain are especially important for heterochromatin nucleation. Independently, these sequences can nucleate a low level of heterochromatin, and nucleation by the RCE is entirely dependent on their presence. These flanking sequences contain a promoter and an intron that are perfectly conserved within the subfamily. Both transcription and splicing are important for heterochromatin nucleation by other sequences (Buscaino *et al.* 2013; Marina *et al.* 2013; Mutazono *et al.* 2017), suggesting that the RCE(dg) promoter and intron may be required for heterochromatin establishment. Surprisingly, RCE(dh) family members, which lack introns and a known promoter (Lee *et al.* 2013), are also capable of nucleating heterochromatin. RCE(dh) flanking sequences may contain an as-yet-unidentified promoter. Alternatively, RCE(dh) members may establish heterochromatin via a transcription-dependent mechanism without a single strong promoter. The heterochromatin nucleating sequence L5 lacks a single well-characterized promoter but contains multiple transcription start sites distributed throughout the element (Partridge *et al.* 2002; Buscaino *et al.* 2013).

While our data demonstrate that the *RevCen* conserved domain and flanking sequences are required for full heterochromatin nucleation by the RCE, the ability of RCEs to recruit heterochromatin to an ectopic site is robust to endogenous genetic variation, and the levels of H3K9me2 recruited to the ectopic site are similar across all RCEs. Differences in H3K9me2 recruitment may be masked by technical and biological variation or, perhaps, by the nature of the ectopic heterochromatin assay. Formation of ectopic heterochromatin domains may reduce yeast fitness and impose an artificial upper limit on the amount of H3K9me2 recruited to the ectopic site. Such a limit could obscure differences in nucleating capacity. This limitation seems unlikely to factor strongly in this particular study, because we have shown that increased levels of H3K9me2, as a consequence of increased Swi6 dosage, can be established at the ectopic site (Wheeler *et al.* 2009). Nonetheless, while the magnitude of H3K9me2 enrichment may lie beyond the dynamic range of our assay, our data demonstrate that all known RCEs are capable of nucleating heterochromatin.

For RCEs to nucleate heterochromatin at an ectopic site, they must recruit heterochromatin assembly machinery in a sequence-dependent manner. The parameters that govern sequence-dependent recruitment often involve the RNAi pathway, and siRNA abundance is a predictor of nucleation ability (Volpe *et al.* 2002, 2003; Buscaino *et al.* 2013). The RNAi pathway may be well suited to establishing heterochromatin in a sequence-flexible manner. Synthetic hairpin RNAs can serve as substrates for Dcr1, with the resulting siRNAs able to target homologous noncentromeric sequences for heterochromatin formation and secondary siRNA production (Iida *et al.* 2008; Simmer *et al.* 2010). Furthermore, siRNAs produced from centromeric transgenes can target a noncentromeric copy of the gene in *trans* (Iida *et al.* 2008; Simmer *et al.* 2010; Kowalik *et al.* 2015; He *et al.* 2016; Yu *et al.* 2018). These results demonstrate that there are not strict sequence requirements for siRNA-mediated heterochromatin formation. Additionally, while nucleating sequences are required for the establishment of heterochromatin at ectopic sites, they are dispensable for its maintenance (Wheeler *et al.* 2012). This study suggests that, even in the context of ectopic heterochromatin domains, sequence-dependent establishment events can be amplified by downstream sequence-independent mechanisms. As long as mutations preserve the initial targeting of a sequence or transcript by the RNAi pathway, sequence-independent mechanisms could act to reinforce heterochromatin. The system established here sets the stage for future studies that use engineered RCEs to directly identify sequence constraints in the nucleation of heterochromatin. These studies can also address the extent of sequence flexibility in an unbiased fashion—the current study may overestimate sequence flexibility within RCEs by selecting for endogenous variants that are compatible with viability.

### Redundancy within centromeres

The *S. pombe* centromere contains heterochromatin domains that are composed of repeat pairs—at least one *dg*/*dh* pair is present on each side of the three *S. pombe* centromeres. As most heterochromatin domains (on either the left or right side of the three centromeres) contain multiple RCEs, we conclude that multiple sequences contribute to heterochromatin establishment within these domains. The left side of centromere 1 and the right side of centromere 2 contain only one RCE. However, the *dg* repeat on the left side of chromosome 1 contains two fragments that can nucleate heterochromatin; one fragment contains RCE(1Ldg), and potentially additional nucleating sequences, and the other fragment is the nucleating sequence L5. Thus, this heterochromatin domain contains at least two nucleating sequences. Furthermore, a version of L5 also exists on the right side of centromere 2. If this copy of L5 is functional, then each heterochromatin domain contains multiple nucleating sequences. This redundancy, coupled with the ability of heterochromatin to spread large distances from a single nucleating sequence (Partridge *et al.* 2000; Wheeler *et al.* 2009), may buffer centromere function from the impact of mutations. While it is not known whether the loss of an individual nucleating sequence has any consequence in the context of an endogenous centromere, deletions of centromere repeats are unstable in *S. pombe* (Chikashige *et al.* 1989), Buscaino *et al.* (2013) have shown that individual nucleating sequences are sufficient for centromere function in the context of an artificial chromosome.

### Like fission yeast centromeres, human centromeres reflect the combined effort of sequence-independent and sequence-dependent pathways

In humans, as in fission yeast, centromeres are chromatin structures built on top of repetitive DNA sequences; human centromeres form on arrays of alpha satellites. Artificial recruitment of CENP-A is sufficient to form a kinetochore (Barnhart *et al.* 2011), and functional neocentromeres can form over noncentromeric, nonrepetitive DNA (Voullaire *et al.* 1993). These findings have led to our understanding of human centromeres as structures that are fundamentally epigenetic. However, studies indicate the existence of parallel sequence-dependent pathways that establish centromere identity (Fachinetti *et al.* 2015; Logsdon *et al.* 2019). Centromere formation in the context of human artificial chromosomes requires higher-order arrays of alpha-satellite sequences and CENP-B boxes, the 17 base-pair binding site for the centromere protein CENP-B (Harrington *et al.* 1997; Ohzeki *et al.* 2002; Hayden *et al.* 2013). Both artificial alphoid DNA lacking CENP-B boxes and alpha satellite from the human Y, which naturally lacks CENP-B boxes, fail to support *de novo* centromere formation. This sequence-dependent mechanism is functionally important in wild-type cells as evidenced by the finding that naturally occurring higher-order repeats that lack CENP-B boxes are associated with higher levels of chromosome missegregation (Fachinetti *et al.* 2015).

The peril of sequence-dependent centromere pathways in both yeast and humans is the lability of centromeric DNA. Centromere repeats are subjected to repeated cycles of mutation, amplification, and homogenization that can result in rapid sequence turnover (Hartley and O’Neill 2019). Defining how these mutagenic processes shape centromere structure and function—and the extent to which they can be accommodated by flexibility, redundancy, and the existence of parallel sequence-independent pathways—is critical for understanding the enigmatic and essential centromere.
